# An integrated host vulnerability score is associated with survival in advanced NSCLC treated with nivolumab: a PET/CT-based real-world study

**DOI:** 10.1186/s12890-026-04496-5

**Published:** 2026-07-16

**Authors:** Vali Aliyev, Murad Guliyev, Mehmet Cem Fidan, Zeliha Birsin, Murat Günaltılı, Emir Çerme, Selin Cebeci, Hamza Abbasov, Said Erkam Bıyıkoğlu, Onur Erdem Şahin, Muhammet Sait Sağer, Nebi Serkan Demirci, Özkan Alan

**Affiliations:** 1 Division of Medical Oncology, Department of Internal Medicine, Cerrahpaşa Faculty of Medicine, Istanbul University-Cerrahpaşa, Istanbul, Türkiye; 2 Department of Nuclear Medicine, Cerrahpaşa Faculty of Medicine, Istanbul University-Cerrahpaşa, Istanbul, 34098 Türkiye

**Keywords:** NSCLC, Immunotherapy, Nivolumab, Host vulnerability, Sarcopenia, Inflammation, Survival

## Abstract

**Background:**

Clinical outcomes in advanced non-small cell lung cancer (NSCLC) treated with immune checkpoint inhibitors remain heterogeneous, and simple prognostic tools for patient stratification are limited. We aimed to evaluate a multidimensional host vulnerability score integrating systemic inflammation, nutritional status, and skeletal muscle depletion in patients receiving second-line nivolumab in a real-world setting.

**Methods:**

In this retrospective study, 110 patients with advanced NSCLC treated with second-line nivolumab were included. A composite score was constructed using neutrophil-to-lymphocyte ratio (NLR), serum albumin, and skeletal muscle index (SMI) derived from pretreatment PET/CT. Patients were stratified into lower-risk (score 0–1) and higher-risk (score 2–3) groups. Survival outcomes were assessed using Kaplan–Meier analysis, Cox proportional hazards models, and model discrimination analyses.

**Results:**

High-risk patients had significantly shorter progression-free survival (median 3.4 vs. 5.9 months; HR 1.68, 95% CI 1.10–2.59; *p* = 0.017) and overall survival (median 7.2 vs. 14.5 months; HR 2.08, 95% CI 1.30–3.31; *p* = 0.002) compared with the low-risk group. In model comparison analyses, the ordinal composite score showed the clearest improvement in prognostic discrimination for overall survival, whereas its incremental value for progression-free survival was more modest.

**Conclusion:**

A multidimensional host vulnerability score integrating inflammatory, nutritional, and body composition domains was associated with survival outcomes in advanced NSCLC treated with nivolumab. This score may support real-world prognostic risk stratification, particularly for overall survival, but requires prospective and external validation before clinical implementation.

**Supplementary Information:**

The online version contains supplementary material available at https://doi.org/10.1186/s12890-026-04496-5.

## Introduction

Lung cancer remains the leading cause of cancer-related mortality worldwide, and non-small cell lung cancer (NSCLC) accounts for approximately 85% of all lung cancer cases [1, 2]. Despite advances in systemic therapy, a substantial proportion of patients present with advanced or metastatic disease, where long-term survival remains limited [3, 4]. In recent years, immune checkpoint inhibitors targeting the programmed death-1 (PD-1) pathway have significantly improved outcomes in advanced NSCLC and have become an important component of modern systemic therapy [5, 6].

Among these agents, nivolumab has demonstrated superior survival compared with docetaxel in previously treated patients with advanced NSCLC, establishing PD-1 blockade as an important treatment option in the second-line setting [5, 7]. Nevertheless, clinical outcomes among patients receiving nivolumab remain heterogeneous. While some patients experience durable responses, others develop early disease progression. This variability has increased interest in identifying simple and clinically accessible prognostic markers that may help improve patient stratification in routine clinical practice [8–10].

Host-related factors reflecting systemic inflammation and nutritional status have emerged as potential determinants of cancer outcomes. The neutrophil-to-lymphocyte ratio (NLR) is a widely available marker of systemic inflammatory response and has been associated with poorer survival across several malignancies, including NSCLC treated with immunotherapy [11, 12]. Similarly, hypoalbuminemia reflects both inflammatory burden and impaired nutritional status and has been linked to unfavorable prognosis in patients with advanced cancer [13]. In addition, inflammatory and nutritional biomarkers have been shown to predict treatment response in other tumor types, including breast cancer [14].

In parallel, body composition parameters have gained increasing attention in oncology. Reduced skeletal muscle mass, often assessed using skeletal muscle index (SMI), reflects cancer-related metabolic and inflammatory alterations and has been associated with adverse outcomes in several malignancies [15]. Emerging evidence suggests that skeletal muscle depletion may also influence the effectiveness of immune checkpoint inhibitors [16], and similar associations between low skeletal muscle index (SMI) and treatment outcomes have been reported in other tumor settings [17].

Although inflammatory markers, nutritional parameters, and body composition metrics have individually been associated with outcomes in patients receiving immune checkpoint inhibitors, their combined prognostic impact remains unclear in patients treated with second-line nivolumab in real-world settings. Because systemic inflammation, nutritional impairment, and skeletal muscle depletion represent interconnected processes reflecting host vulnerability, integrating these variables may provide a more comprehensive prognostic assessment. Therefore, this study aimed to evaluate the prognostic value of a composite inflammatory–nutritional–skeletal muscle score based on NLR, serum albumin level, and skeletal muscle index (SMI) in patients receiving immune checkpoint inhibitor therapy. Real-world data on integrated host-related prognostic indices in patients receiving immunotherapy remain limited, particularly in single-center clinical settings.

## Materials and methods

### Study design and patient population

This retrospective cohort study included patients with advanced NSCLC who received nivolumab at the Department of Medical Oncology, İstanbul University-Cerrahpaşa, Cerrahpaşa Faculty of Medicine. Patients treated between January 2018 and June 2025 were screened for eligibility.

Eligible patients had histologically confirmed NSCLC and received nivolumab as second-line therapy following progression on platinum-based chemotherapy. To maintain treatment homogeneity, patients receiving other PD-1/PD-L1 inhibitors were not included. Patients were required to have baseline laboratory data obtained prior to nivolumab initiation and pretreatment PET/CT imaging suitable for body composition analysis. Baseline laboratory parameters were obtained within 14 days before nivolumab initiation. Pretreatment PET/CT was defined as the closest available PET/CT performed before nivolumab initiation. Patients harboring known actionable oncogenic driver alterations (including EGFR mutations, ALK rearrangements, and ROS1 fusions), incomplete clinical data, unavailable imaging for skeletal muscle assessment, or evidence of active infection at the time of baseline laboratory evaluation were excluded.

Clinical variables including age, sex, smoking status, ECOG performance status, histological subtype, metastatic sites, and prior treatments were retrieved from electronic medical records. All clinical and laboratory data were reviewed and verified by the study investigators.

### PET/CT imaging and skeletal muscle assessment

Baseline PET/CT scans were used, and skeletal muscle measurements were derived from the CT component. Patients fasted for at least 6 h, with blood glucose levels < 200 mg/dL prior to imaging. A weight-based dose of ^18F-FDG (3.5–5.0 MBq/kg) was administered, followed by a 50–60 min uptake period. Imaging was performed from the skull vertex to mid-thigh using a dedicated PET/CT system (Discovery 710, GE Healthcare, Milwaukee, WI, USA).

**Fig. 1:**
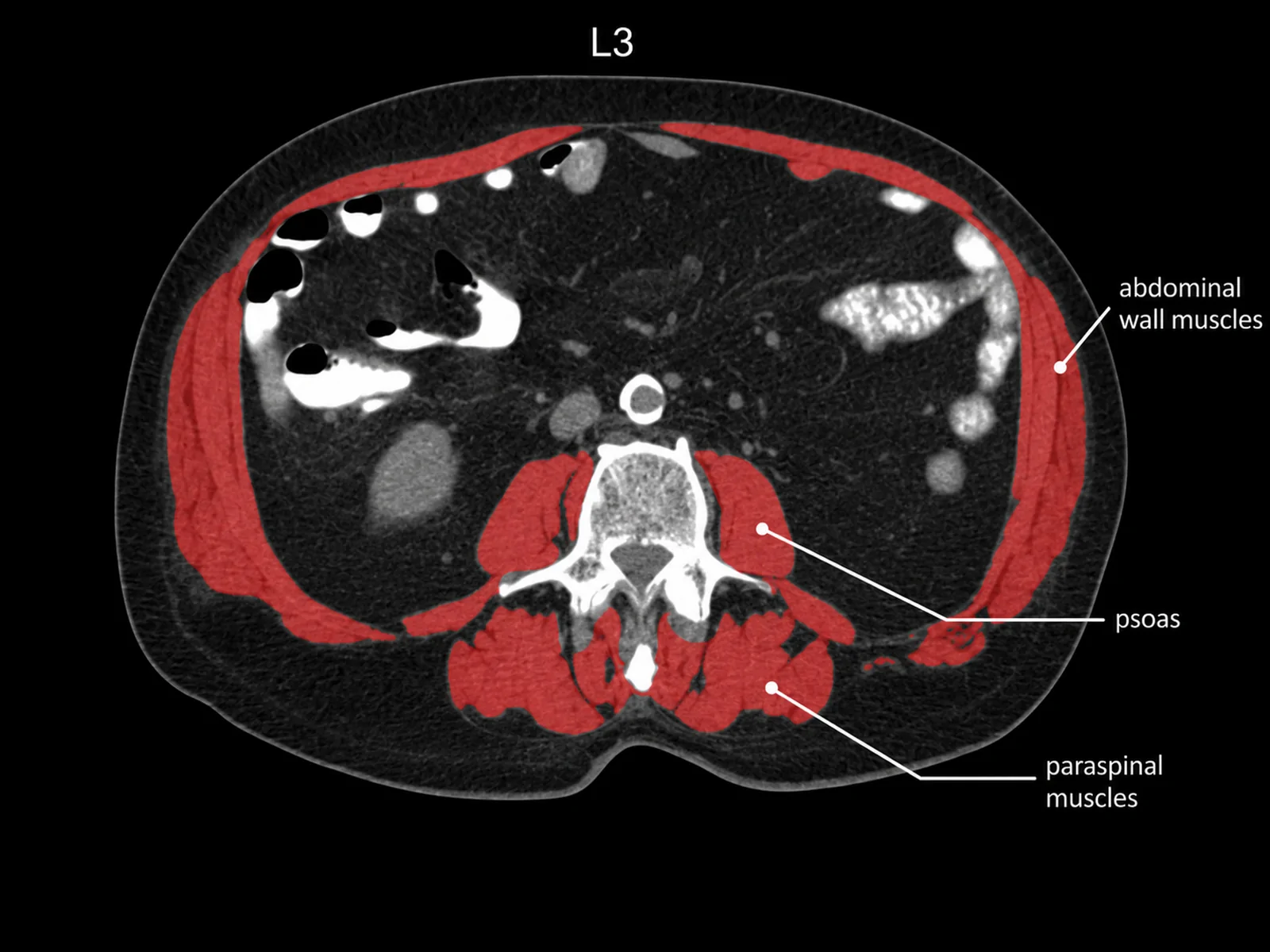
Representative L3 skeletal muscle segmentation on axial CT. Axial CT image at the level of the third lumbar vertebra (L3) showing segmentation of the skeletal muscle area used for skeletal muscle index (SMI) assessment. The highlighted regions include the psoas, paraspinal, and abdominal wall muscles. Skeletal muscle area was measured using predefined Hounsfield unit thresholds (− 29 to + 150 HU) and normalized for height squared to calculate SMI

Images were reconstructed using an OSEM iterative algorithm (3 iterations, 24 subsets) with a 5-mm Gaussian filter and a 256 × 256 matrix. Skeletal muscle area (SMA) was measured at the level of the third lumbar vertebra (L3) on axial CT images using standard Hounsfield unit thresholds (− 29 to + 150 HU). The skeletal muscle index (SMI, cm²/m²) was calculated by normalizing SMA to height squared (Fig. 1). Skeletal muscle measurements were evaluated by two investigators blinded to survival outcomes. Interobserver reproducibility was formally assessed in a randomly selected subset of 30 patients using the intraclass correlation coefficient based on a two-way random-effects model for absolute agreement.

An exploratory metabolic parameter, the metabolic muscle ratio (MMR), was also assessed, defined as the ratio of psoas muscle SUVmean to liver SUVmean. Patients were stratified into low and high MMR groups using the cohort median. Low SMI was defined using sex-specific thresholds (< 52 cm²/m² for men and < 38 cm²/m² for women) based on previously reported cut-offs [18, 19].

### Laboratory parameters

Baseline laboratory values obtained prior to nivolumab initiation were collected from routine blood tests. NLR was calculated as: NLR = neutrophil count (10³/mm³) / lymphocyte count (10³/mm³), reflecting systemic inflammatory status in cancer patients [11]. Serum albumin levels were used as a marker of nutritional status. Values below 3.5 g/dL, corresponding to the lower limit of the normal reference range, were defined as hypoalbuminemia.

### Composite inflammatory–nutritional–skeletal muscle score

**Fig. 2:**
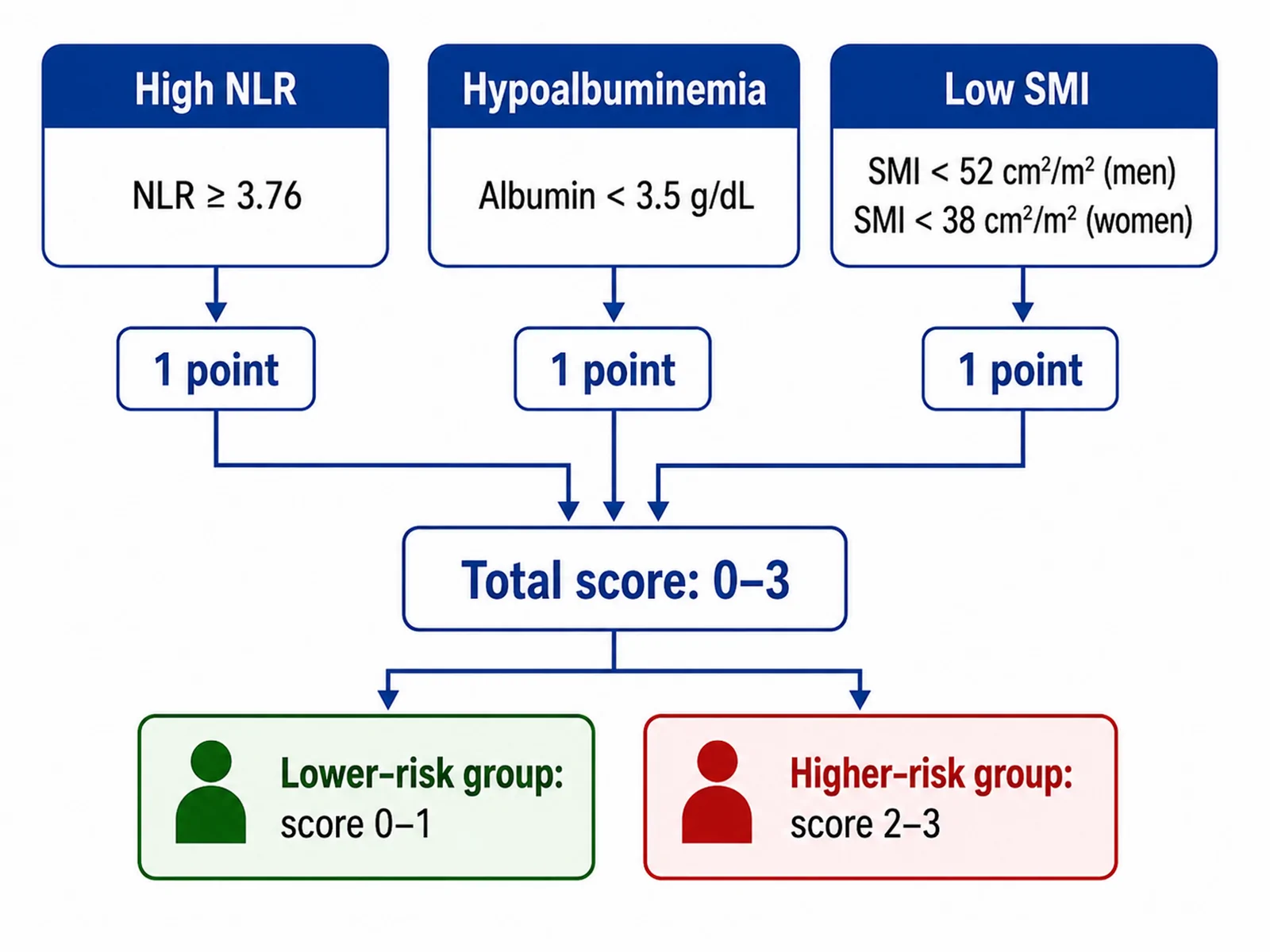
Composite host vulnerability score. The score was constructed using three host-related parameters: high neutrophil-to-lymphocyte ratio (NLR ≥ 3.76), hypoalbuminemia (albumin < 3.5 g/dL), and low skeletal muscle index (SMI < 52 cm²/m² for men and < 38 cm²/m² for women). Each parameter contributed 1 point, yielding a total score ranging from 0 to 3. Patients were subsequently classified into a lower-risk group (score 0–1) or a higher-risk group (score 2–3)

A composite score integrating systemic inflammation, nutritional status, and skeletal muscle depletion was constructed using NLR, serum albumin level, and SMI. Each adverse parameter was assigned one point, resulting in a total score ranging from 0 (no adverse factors) to 3 (all three adverse factors present). Receiver operating characteristic (ROC) curve analysis did not identify a clinically meaningful or statistically robust cut-off value for NLR; therefore, the cohort median was used as a pragmatic threshold to avoid arbitrary categorization. Patients received one point for NLR ≥ 3.76, corresponding to the cohort median, one point for serum albumin < 3.5 g/dL, and one point for low SMI. For survival analyses, patients were categorized into low-risk (score 0–1) and high-risk (score 2–3) groups (Fig. 2).

### Outcome assessment and follow-up

Disease progression was assessed based on radiological evaluation using cross-sectional imaging performed during routine clinical follow-up. Imaging assessments were conducted at intervals of approximately 8 − 12 weeks in accordance with our institutional practice. Disease progression was investigator-assessed based on routine radiologic evaluation and clinical documentation. Central radiologic review was not performed.

Progression-free survival (PFS) was defined as the interval between the first nivolumab administration and the occurrence of either radiologic disease progression or death from any cause, whichever occurred first. Patients without documented progression were censored at their most recent follow-up assessment. Overall survival (OS) was defined as the time from the first nivolumab dose to death from any cause. Patients who were alive at the time of analysis were censored at the date of their last follow-up.

### Adverse events

Treatment-related adverse events were extracted from medical records and graded according to the National Cancer Institute Common Terminology Criteria for Adverse Events (CTCAE) version 5.0 [20].

### Statistical analysis

All analyses were performed using IBM SPSS Statistics version 27.0 (IBM Corp., Armonk, NY, USA). Continuous variables were summarized as median (interquartile range [IQR] ) and categorical variables were presented as frequencies and percentages. Survival curves were estimated using the Kaplan–Meier method, and differences between groups were compared using the log-rank test. Univariable and multivariable Cox proportional hazards regression analyses were performed to identify factors associated with survival outcomes. Variables with *p* value < 0.10 in univariable analysis were included in the multivariable model. Hazard ratios (HRs) and 95% confidence intervals (CIs) were reported. A two-sided *p* value < 0.05 was considered statistically significant. Model discrimination was further assessed using Harrell’s C-index and Akaike information criterion (AIC). A base clinical model including age ≥ 65 years, ECOG performance status ≥ 2, and liver metastasis was compared with models incorporating individual host-related parameters, the albumin–SMI score, the ordinal composite score, and the dichotomized composite risk group. Additional sensitivity analyses evaluated patients with available PD-L1 expression data, individual host-related parameters, an NLR-free albumin–SMI score, and a BMI-integrated SMI-based composite score.

### Ethics statement

This study was conducted in accordance with the Declaration of Helsinki and was approved by the institutional ethics committee (Approval No: E-74555795-050.04-1231222; January 22, 2025). Due to the retrospective nature of the study, the requirement for informed consent was waived. Clinical trial number: not applicable.

## Results

### Study population

A total of 110 patients with advanced non-small cell lung cancer who received second-line nivolumab were included in the analysis. Baseline demographic and clinicopathologic characteristics are summarized in Table 1, with additional cohort details provided in Supplementary Table 1.

**Table 1:** Baseline characteristics of the study population according to composite risk score

Characteristic	Overall (*n* = 110)	Lower-risk (*n* = 73)	Higher-risk (*n* = 37)	*p* value
Age, years — median (IQR)	64.5 (58–70)	64.0 (57–70)	66.5 (59–70)	0.61
Age ≥ 65 years, no. (%)	55 (50.0)	35 (47.9)	20 (54.1)	0.54
Male sex, no. (%)	80 (72.7)	51 (69.9)	29 (78.4)	0.36
Smoking history, no. (%)				0.81
Yes	89 (80.9)	59 (80.8)	30 (81.1)	
ECOG performance status, no. (%)				0.31
0	10 (9.1)	8 (11.0)	2 (5.4)	
1	93 (84.5)	61 (83.6)	32 (86.5)	
≥2	7 (6.4)	4 (5.5)	3 (8.1)	
Histologic subtype, no. (%)				0.29
Adenocarcinoma	67 (60.9)	46 (63.0)	21 (56.8)	
Squamous cell carcinoma	38 (34.5)	25 (34.2)	13 (35.1)	
Not otherwise specified	5 (4.5)	2 (2.7)	3 (8.1)	
PD-L1 expression, no. (%)				0.92
<1%	19 (17.3)	13 (17.8)	6 (16.2)	
≥1%	41 (37.3)	27 (37.0)	14 (37.8)	
Unknown	50 (45.5)	33 (45.2)	17 (46.0)	
Metastatic disease timing, no. (%)				0.96
Synchronous	59 (53.6)	39 (53.4)	20 (54.1)	
Metachronous	51 (46.4)	34 (46.6)	17 (45.9)	
Metastatic sites, no. (%)				
Bone metastasis	40 (36.4)	26 (35.6)	14 (37.8)	0.82
Brain metastasis	20 (18.2)	15 (20.5)	5 (13.5)	0.37
Liver metastasis	18 (16.4)	12 (16.4)	6 (16.2)	1.00
≥ 2 extrathoracic metastatic organs, no. (%)	27 (24.5)	16 (21.9)	11 (29.7)	0.38

*Abbreviations*: *ECOG* Eastern Cooperative Oncology Group, *PD-L1* programmed death-ligand 1, *IQR* interquartile range

The median age of the cohort was 64.5 years (IQR 58–70), and 80 patients (72.7%) were male. Adenocarcinoma represented the most common histologic subtype (60.9%), followed by squamous cell carcinoma (34.5%). Most patients had good performance status at treatment initiation, with 93 patients (84.5%) having ECOG performance status 1.

Synchronous metastatic disease was observed in 59 patients (53.6%), whereas 51 patients (46.4%) developed metachronous metastases. Bone metastases were present in 40 patients (36.4%), brain metastases in 20 patients (18.2%), and liver metastases in 18 patients (16.4%). Baseline characteristics did not differ significantly between the low-risk and high-risk groups across measured variables (all *p* > 0.05), indicating a comparable distribution of clinical features.

### Follow-up and event distribution

The median follow-up duration was 18.4 months (IQR 10–31). During the follow-up period, 92 progression events (83.6%) and 79 death events (71.8%) were observed in the study cohort. The median PFS and OS for the overall cohort were 4.2 and 11.8 months, respectively.

### Distribution of composite risk groups

According to the predefined composite inflammatory–nutritional–skeletal muscle score, 73 patients (66.4%) were classified as low risk and 37 patients (33.6%) as high risk. The distribution of composite scores within the study cohort is shown in Supplementary Table 3.

### Survival outcomes according to composite risk group

**Fig. 3:**
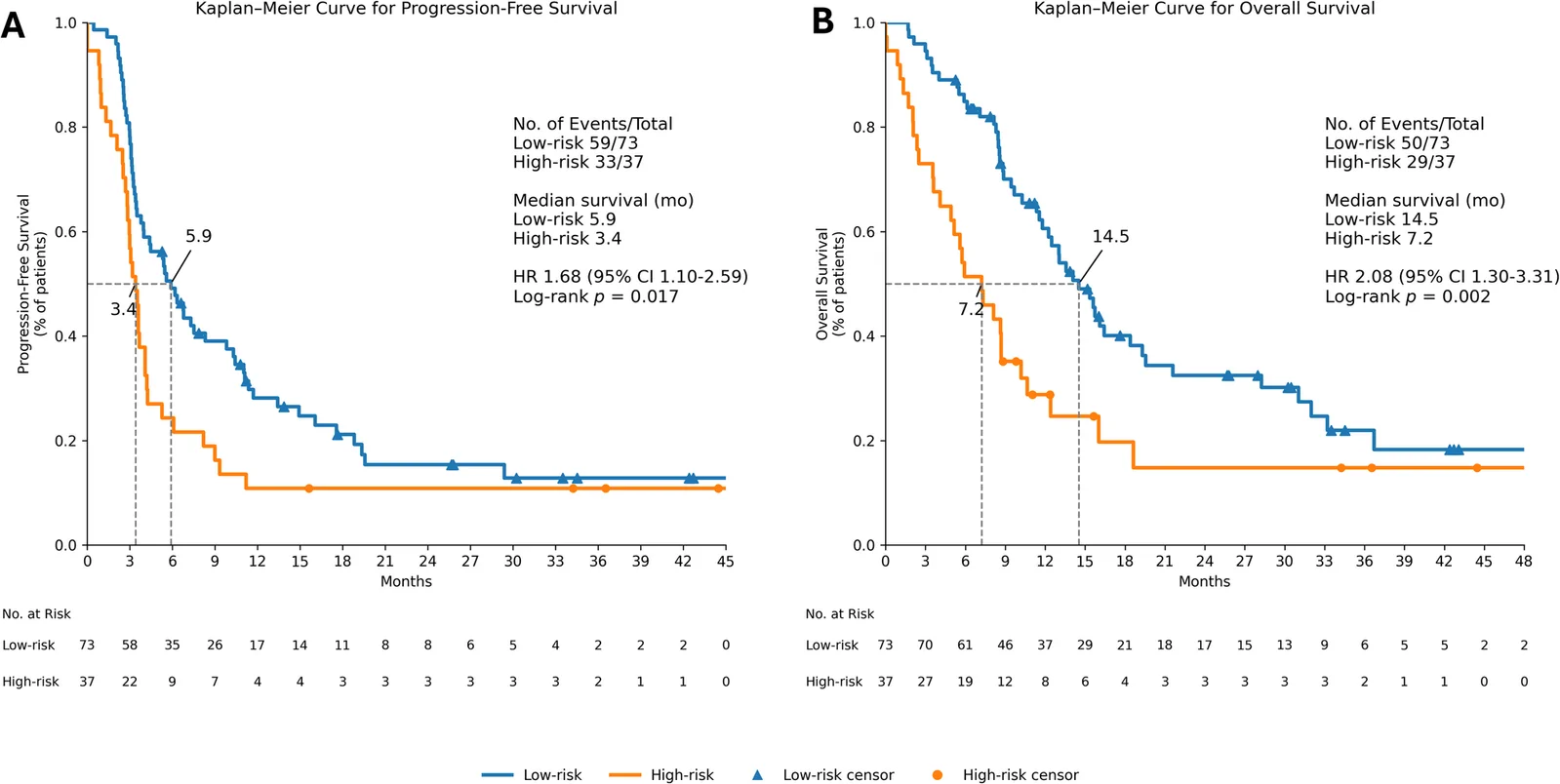
Kaplan–Meier survival curves according to composite host vulnerability score. **A** Progression-free survival (PFS) and (**B**) overall survival (OS) stratified by low-risk (score 0–1) and high-risk (score 2–3) groups. High-risk patients demonstrated significantly shorter PFS (median 3.4 vs. 5.9 months; HR 1.68, 95% CI 1.10–2.59; log-rank *p* = 0.017) and OS (median 7.2 vs. 14.5 months; HR 2.08, 95% CI 1.30–3.31; log-rank *p* = 0.002). Hazard ratios shown in the Kaplan–Meier plots were derived from univariable Cox proportional hazards models

Kaplan–Meier survival analysis demonstrated significantly different survival outcomes between the two composite risk groups (Fig. 3). For progression-free survival, disease progression or death occurred in 59 of 73 patients in the low-risk group and 33 of 37 patients in the high-risk group. Median PFS was 5.9 months in the low-risk group and 3.4 months in the high-risk group. High composite risk was associated with a significantly increased risk of progression or death (HR 1.68, 95% CI 1.10–2.59; log-rank *p* = 0.017) (Fig. 3A).

For overall survival, death occurred in 50 of 73 patients in the low-risk group and 29 of 37 patients in the high-risk group. Median OS was 14.5 months in the low-risk group and 7.2 months in the high-risk group. High composite risk was associated with significantly worse overall survival (HR 2.08, 95% CI 1.30–3.31; log-rank *p* = 0.002) (Fig. 3B).

Additional Kaplan–Meier analyses exploring survival according to individual host-related factors are presented in the Supplementary Material. Survival outcomes stratified by SMI status, NLR, serum albumin level, and exploratory MMR are shown in Supplementary Figs. 1–4, respectively. Furthermore, survival across the full composite score (0–3) is illustrated in Supplementary Fig. 5.

### Cox regression analyses

For progression-free survival, univariable Cox regression analysis identified age ≥ 65 years, ECOG performance status ≥ 2, liver metastasis, hypoalbuminemia, and the composite risk group as factors associated with worse outcomes. High composite risk was associated with shorter progression-free survival in univariable analysis (HR 1.68, 95% CI 1.10–2.59; *p* = 0.017). In the multivariable model adjusting for age, ECOG performance status, and liver metastasis, high composite risk remained independently associated with shorter progression-free survival (HR 2.06, 95% CI 1.33–3.21; *p* = 0.001).

For overall survival, univariable Cox regression analysis identified ECOG performance status ≥ 2, liver metastasis, ≥ 2 extrathoracic metastatic organs, NLR ≥ 3.76, low SMI, hypoalbuminemia, and the composite risk group as factors associated with increased mortality. High composite risk was associated with worse overall survival in univariable analysis (HR 2.08, 95% CI 1.30–3.31; *p* = 0.002). After adjustment for age, ECOG performance status, and liver metastasis, high composite risk remained independently associated with poorer overall survival (HR 2.48, 95% CI 1.54–4.01; *p* < 0.001).

**Fig. 4:**
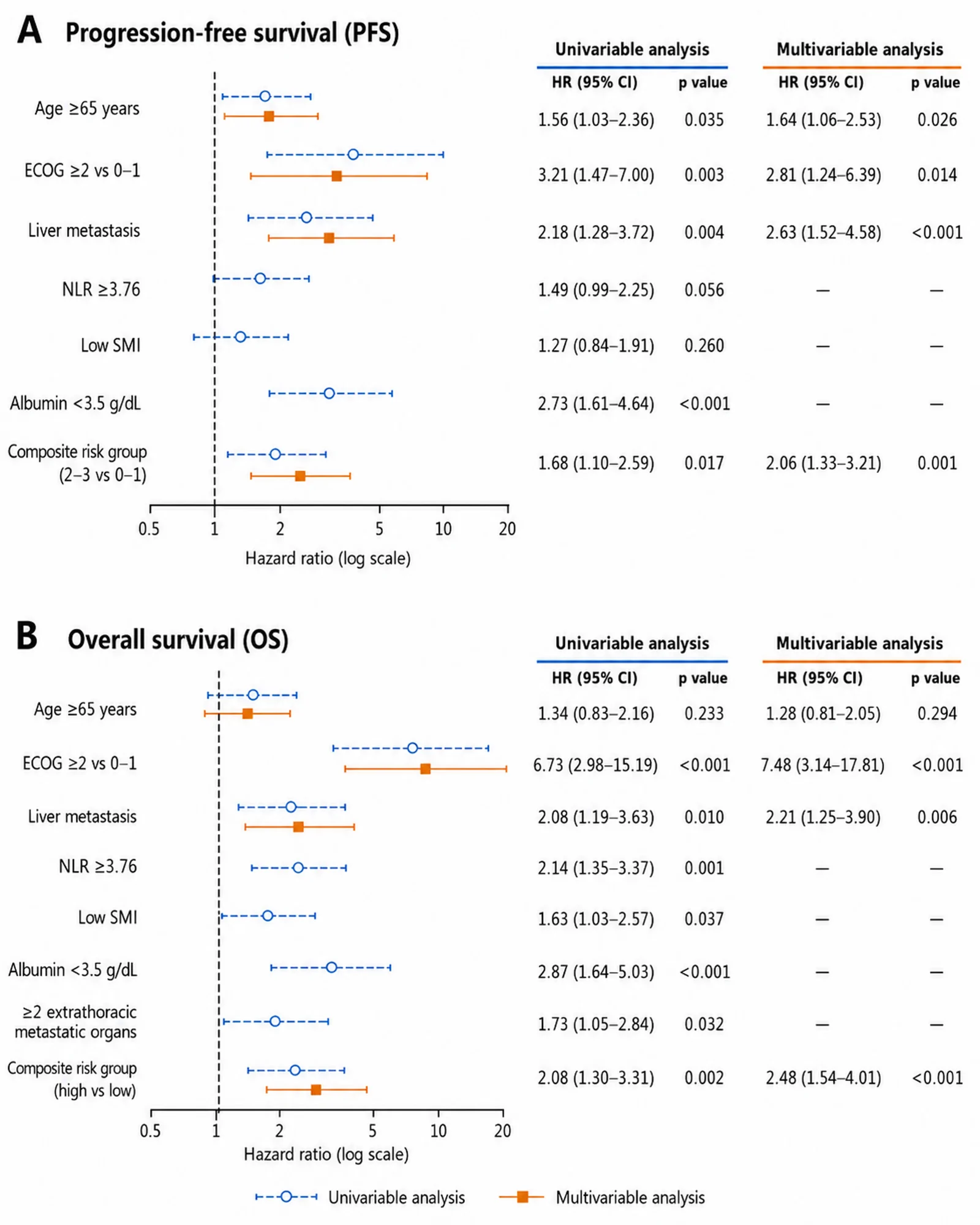
Univariable and multivariable Cox proportional hazards analyses for survival outcomes. **A** Progression-free survival (PFS) and (**B**) overall survival (OS). Open circles with dashed lines represent univariable estimates, and filled squares with solid lines represent multivariable estimates. HR, hazard ratio; CI, confidence interval; ECOG, Eastern Cooperative Oncology Group; NLR, neutrophil-to-lymphocyte ratio; SMI, skeletal muscle index

A similar modeling strategy was applied, and individual components were not included to avoid multicollinearity. The results of univariable and multivariable Cox regression analyses for progression-free and overall survival are summarized in Tables 2 and 3 and visually presented in Fig. 4. Continuous Cox regression analyses for host-related variables are presented in Supplementary Table 4. In exploratory analyses, MMR evaluated as a continuous variable was associated with overall survival; however, this association was accompanied by extremely wide confidence intervals, indicating limited precision and potential instability of the estimate.

**Table 2:** Univariable and multivariable Cox regression analysis for progression-free survival

Variable	Univariable HR (95% CI)	*p*	Multivariable HR (95% CI)	*p*
Age ≥ 65 years	1.56 (1.03–2.36)	0.035	1.64 (1.06–2.53)	0.026
Male sex	0.83 (0.52–1.31)	0.417	—	—
ECOG ≥ 2 vs. 0–1	3.21 (1.47–7.00)	0.003	2.81 (1.24–6.39)	0.014
Adenocarcinoma vs. non-adenocarcinoma	0.92 (0.60–1.40)	0.693	—	—
Synchronous vs. metachronous disease	1.13 (0.75–1.71)	0.558	—	—
Bone metastasis	1.39 (0.91–2.12)	0.129	—	—
Brain metastasis	0.94 (0.55–1.61)	0.808	—	—
Liver metastasis	2.18 (1.28–3.72)	0.004	2.63 (1.52–4.58)	< 0.001
≥ 2 extrathoracic metastatic organs	1.32 (0.82–2.12)	0.249	—	—
NLR ≥ 3.76	1.49 (0.99–2.25)	0.056	—	—
Low SMI	1.27 (0.84–1.91)	0.260	—	—
Albumin < 3.5 g/dL	2.73 (1.61–4.64)	< 0.001	—	—
Composite risk group (2–3 vs. 0–1)	1.68 (1.10–2.59)	0.017	2.06 (1.33–3.21)	0.001
Composite score (per 1-point increase)	1.51 (1.17–1.93)	0.001	—	—
MMR (high vs. low)	0.96 (0.64–1.45)	0.855	—	—
BMI (continuous)	0.99 (0.97–1.01)	0.344	—	—

*Abbreviations*: *HR* hazard ratio, *CI* confidence interval, *ECOG* Eastern Cooperative Oncology Group, *NLR* neutrophil-to-lymphocyte ratio, *SMI* skeletal muscle index, *BMI* body mass index, *MMR* metabolic muscle ratio

**Table 3:** Univariable and multivariable Cox regression analysis for overall survival

Variable	Univariable HR (95% CI)	*p*	Multivariable HR (95% CI)	*p*
Age ≥ 65 years	1.34 (0.83–2.16)	0.233	1.28 (0.81–2.05)	0.294
Male sex	1.01 (0.60–1.67)	0.984	—	—
ECOG ≥ 2 vs. 0–1	6.73 (2.98–15.19)	< 0.001	7.48 (3.14–17.81)	< 0.001
Adenocarcinoma vs. non-adenocarcinoma	0.81 (0.51–1.29)	0.372	—	—
Synchronous vs. metachronous disease	1.01 (0.64–1.59)	0.965	—	—
Bone metastasis	1.52 (0.96–2.40)	0.075	—	—
Brain metastasis	0.94 (0.53–1.68)	0.839	—	—
Liver metastasis	2.08 (1.19–3.63)	0.010	2.21 (1.25–3.90)	0.006
≥ 2 extrathoracic metastatic organs	1.73 (1.05–2.84)	0.032	—	—
NLR ≥ 3.76	2.14 (1.35–3.37)	0.001	—	—
Low SMI	1.63 (1.03–2.57)	0.037	—	—
Albumin < 3.5 g/dL	2.87 (1.64–5.03)	< 0.001	—	—
Composite risk group (high vs. low)	2.08 (1.30–3.31)	0.002	2.48 (1.54–4.01)	< 0.001
Composite score (per 1-point increase)	1.93 (1.46–2.54)	< 0.001	—	—
MMR (high vs. low)	0.92 (0.58–1.46)	0.732	—	—
BMI (continuous)	0.97 (0.92–1.02)	0.232	—	—

*Abbreviations*: *HR* hazard ratio, *CI* confidence interval, *ECOG* Eastern Cooperative Oncology Group, *NLR* neutrophil-to-lymphocyte ratio, *SMI* skeletal muscle index, *BMI* body mass index, *MMR* metabolic muscle ratio

Model comparison analyses were performed to compare the composite score with individual host-related parameters. For progression-free survival, the ordinal composite score modestly improved discrimination compared with the clinical model alone, although the albumin-only model had the lowest AIC. For overall survival, the ordinal composite score showed the highest C-index and lowest AIC among the evaluated models (Supplementary Table 5).

In a sensitivity analysis restricted to patients with available PD-L1 expression data (*n* = 60), the association between high composite risk and worse outcomes was preserved for both progression-free survival and overall survival. High composite risk remained associated with shorter PFS and OS after adjustment for clinical variables (Supplementary Table 6).

Additional sensitivity analyses showed that the NLR-free albumin–SMI score and the BMI-integrated composite score remained associated with both progression-free and overall survival. In separate adjusted models, albumin and high NLR retained independent associations, whereas low SMI alone did not remain independently associated after adjustment (Supplementary Table 7).

Interobserver reproducibility for SMI measurement was excellent in the randomly selected subset of 30 patients, with an intraclass correlation coefficient of 0.973 (Supplementary Table 8).

### Treatment-related adverse events

Nivolumab-related adverse events were observed in 16 patients (14.5%). Most toxicities were grade 2, occurring in 10 patients (9.1%), while grade ≥ 3 adverse events occurred in 6 patients (5.5%). Treatment interruption or permanent discontinuation due to toxicity occurred in 11 patients (10.0%). A detailed distribution of immune-related adverse events is provided in Supplementary Table 9.

## Discussion

In this retrospective real-world cohort of patients with advanced NSCLC treated with second-line nivolumab, we investigated the prognostic significance of a composite score integrating systemic inflammation, nutritional status, and skeletal muscle depletion. Our findings demonstrated that patients classified as high risk according to this composite host-related score experienced significantly worse progression-free and overall survival compared with those in the low-risk group. Importantly, the prognostic value of the composite score remained significant after adjustment for established clinical factors such as ECOG performance status and liver metastasis. Liver metastasis also remained independently associated with worse outcomes, whereas bone metastasis was not retained in the final model, suggesting that the score should be interpreted together with conventional disease-burden variables. These results suggest that host-related biological characteristics may contribute to outcome variability among patients receiving immune checkpoint inhibitor therapy.

Systemic inflammatory status has increasingly been recognized as an important determinant of outcomes during immunotherapy. Several studies conducted in patients with advanced NSCLC treated with PD-1 blockade have shown that elevated baseline inflammatory markers, particularly the neutrophil-to-lymphocyte ratio, are associated with inferior survival outcomes. Clinical analyses from immunotherapy-treated cohorts have reported that patients with higher baseline NLR values tend to experience shorter survival during nivolumab therapy, supporting the potential role of inflammatory biomarkers in prognostic assessment during immune checkpoint inhibitor treatment [21–23]. These findings are biologically plausible, as chronic systemic inflammation may promote tumor progression and impair effective antitumor immune responses.

Beyond inflammatory markers, body composition parameters have also gained increasing attention as determinants of cancer outcomes. Reduced skeletal muscle mass reflects complex metabolic alterations associated with cancer progression, including systemic inflammation, increased catabolism, and impaired energy metabolism. Previous investigations across different malignancies have demonstrated that low skeletal muscle mass is associated with poorer survival outcomes [24–26]. More recently, studies focusing on patients receiving immune checkpoint inhibitors have suggested that reduced skeletal muscle reserves may also be associated with inferior survival during immunotherapy [27–29]. Emerging evidence from more recent analyses further supports the prognostic relevance of body composition parameters in patients undergoing immune checkpoint inhibitor therapy for advanced lung cancer [30, 31]. These findings suggest that skeletal muscle depletion may represent an important host-related factor reflecting impaired physiological reserve and vulnerability to disease progression.

Our study integrates these interrelated host-related domains into a single composite score in patients receiving second-line nivolumab, a clinical setting in which validated prognostic tools remain limited. Formal model comparison showed that the ordinal composite score provided the clearest incremental discrimination for overall survival, whereas its added value for progression-free survival was more modest and hypoalbuminemia alone remained a strong prognostic factor. These findings suggest that the composite score may better reflect longer-term host vulnerability and mortality risk than early disease progression alone. Therefore, the score should be interpreted as a prognostic host-vulnerability measure rather than a treatment-specific predictive biomarker. This integrated approach may better capture the interaction between systemic inflammation, nutritional impairment, and metabolic alterations in advanced disease [32, 33].

Sensitivity analyses suggested that the prognostic signal was not dependent on a single score definition. However, the limited independent effect of SMI alone supports interpreting the composite score as an integrated host-vulnerability measure rather than as evidence that each individual component has equivalent prognostic weight.

From a clinical perspective, this score may help identify patients who require closer monitoring, nutritional assessment, and early supportive-care referral. However, it should not be used to withhold or modify immunotherapy without prospective validation. Consequently, this composite score may represent a practical approach for risk stratification in routine clinical practice [34, 35].

Immune-related adverse events may be associated with outcomes during immune checkpoint inhibitor therapy; however, in this retrospective cohort, adverse events were few and recorded descriptively. Therefore, their time-dependent association with survival and systemic inflammation could not be reliably evaluated.

Exploratory analyses including a PET-derived MMR based on SUVmean yielded inconsistent findings. Although a statistically significant association with overall survival was observed in continuous analysis, the extremely wide confidence intervals indicate limited precision of the estimate, likely reflecting the small sample size and sensitivity to outliers.

Taken together, these findings suggest that a multidimensional assessment of host-related factors may provide additional prognostic information beyond conventional clinical variables, particularly for overall survival. Given that all components are derived from routinely available clinical and imaging data, this approach may represent a practical tool for real-world risk stratification. However, these findings should be interpreted with caution due to the retrospective design, and prospective external validation is required before clinical implementation.

## Limitations

This study has several limitations that should be considered. The retrospective single-center design may introduce selection bias and residual confounding. The relatively limited sample size may have increased the risk of model instability and reduced the precision of effect estimates, particularly in exploratory analyses. The proposed composite score was not externally validated, and therefore its generalizability remains uncertain until confirmed in larger, multicenter cohorts. PD-L1 expression was unavailable for a substantial proportion of patients, limiting comprehensive adjustment for this established immunotherapy-related biomarker. Although a sensitivity analysis restricted to patients with available PD-L1 data showed consistent associations, this subgroup analysis was exploratory and limited by sample size.

Skeletal muscle assessment was based solely on imaging-derived SMI, as data on muscle strength and physical performance were not available; therefore, the term low SMI was used rather than sarcopenia. The SMI cut-offs were derived from Western populations, and population-specific validation is needed despite consistent findings in BMI-integrated sensitivity analyses. Additionally, all host-related parameters were assessed at a single baseline time point, and dynamic changes during treatment were not evaluated. Furthermore, the NLR cut-off was defined using the cohort-specific median. While this approach is commonly used in exploratory analyses, the absence of a standardized threshold may limit comparability across studies. Finally, exploratory analyses including a PET-derived MMR yielded inconsistent results and should be interpreted cautiously, as these findings may reflect limited statistical power and susceptibility to outlier effects.

## Conclusion

In conclusion, a composite score integrating NLR, serum albumin, and skeletal muscle index was associated with survival outcomes in patients with advanced NSCLC treated with nivolumab. This multidimensional host-related assessment may provide additional prognostic information beyond conventional clinical variables, particularly for overall survival. Prospective and multicenter studies are required to validate these findings before clinical implementation.

## Supplementary Information

Supplementary Material 1.

## Data Availability

The datasets supporting the conclusions of this study are included within the article and its supplementary files. Additional data are available from the corresponding author on reasonable request.

## Abbreviations

BMI: Body mass index
CI: Confidence interval
ECOG: Eastern Cooperative Oncology Group
HR: Hazard ratio
IQR: Interquartile range
MMR: Metabolic muscle ratio
NLR: Neutrophil-to-lymphocyte ratio
OS: Overall survival
PFS: Progression-free survival
SMI: Skeletal muscle index
