# Supplementary material for: An integrated host vulnerability score is associated with survival in advanced NSCLC treated with nivolumab: a PET/CT-based real-world study
Source: BMC Pulm Med. 2026 Jul 16;26:397. doi: 10.1186/s12890-026-04496-5 (PMC13543483; doi:10.1186/s12890-026-04496-5)
Supplement: Supplementary file 1 [file 12890_2026_4496_MOESM1_ESM.docx]

**The supplementary material provides additional tables and figures supporting the analyses presented in the main manuscript.**

**Supplementary Table 1.** Baseline demographic, clinicopathologic, and body composition characteristics of the overall study cohort (n = 110).

| **Characteristic** | **Statistic** | **Value** |
| --- | --- | --- |
| Age, years | Median (IQR) | 64.5 (58–70) |
|  | ≥65 years, n (%) | 55 (50.0) |
| Male sex | n (%) | 80 (72.7) |
| Smoking history | Yes, n (%) | 89 (80.9) |
| ECOG performance status | 0, n (%) | 10 (9.1) |
|  | 1 | 93 (84.5) |
|  | ≥2 | 7 (6.4) |
| Histologic subtype | Adenocarcinoma, n (%) | 67 (60.9) |
|  | Squamous cell carcinoma | 38 (34.5) |
|  | NOS | 5 (4.5) |
| PD-L1 expression | <1%, n (%) | 19 (17.3) |
|  | ≥1% | 41 (37.3) |
|  | Unknown | 50 (45.5) |
| Metastatic disease timing | Synchronous, n (%) | 59 (53.6) |
|  | Metachronous | 51 (46.4) |
| Metastatic sites | Bone metastasis, n (%) | 40 (36.4) |
|  | Brain metastasis | 20 (18.2) |
|  | Liver metastasis | 18 (16.4) |
| Disease burden | ≥2 extrathoracic metastatic organs, n (%) | 27 (24.5) |
| Inflammatory markers | NLR, median (IQR) | 3.76 (2.22–6.61) |
| Nutritional status | Albumin, g/dL median (IQR) | 4.0 (3.7–4.25) |
|  | Albumin <3.5 g/dL, n (%) | 18 (16.4) |
| Body composition | BMI, kg/m² median (IQR) | 25.3 (22.8–27.1) |
|  | SMI, cm²/m² median (IQR) | 46.9 (40.4–52.7) |
|  | Low SMI*, n (%) | 59 (53.6) |
| Metabolic muscle marker | MMR, median (IQR) | 0.258 (0.219–0.297) |
| Follow-up | Median follow-up, months (IQR) | 18.4 (10–31) |
| PFS events | n (%) | 92 (83.6) |
| OS events | n(%) | 79 (71.8) |
|  |  |  |

*Low SMI defined as <52 cm²/m² for men and <38 cm²/m² for women.

Continuous variables are presented as median (interquartile range), and categorical variables are presented as number (percentage).

**Supplementary Table 2.** Definition and scoring system of the composite inflammatory–nutritional–skeletal muscle score.

| **Variable** | **Definition** | **Score** |
| --- | --- | --- |
| Skeletal Muscle Index (SMI) | <52 cm²/m² for men and <38 cm²/m² for women | 1 |
| Neutrophil-to-Lymphocyte Ratio (NLR) | ≥ 3.76 | 1 |
| Serum Albumin | <3.5 g/dL | 1 |

**Total score range:** 0–3

Each variable contributed one point to the score. The total composite score ranged from 0 to 3, with higher scores indicating a greater inflammatory, nutritional, and skeletal muscle burden. Patients were categorized into lower-risk (score 0–1) and higher-risk (score 2–3) groups for survival analyses.

**Supplementary Table 3.** Distribution of the composite inflammatory–nutritional– skeletal muscle score in the study cohort (n = 110).

| **Composite Score** | **Patients (n)** | **Percentage (%)** |
| --- | --- | --- |
| 0 | 27 | 24.5 |
| 1 | 46 | 41.8 |
| 2 | 25 | 22.7 |
| 3 | 12 | 10.9 |
| **Total** | **110** | **100** |

**Median composite score (IQR):** 1 (0–2)

**Supplementary Table 4.** Univariable Cox regression analyses of continuous variables for progression-free survival and overall survival.

| **Variable** | **PFS HR (95% CI)** | ***p*  value** | **OS  HR (95% CI)** | ***p*  value** |
| --- | --- | --- | --- | --- |
| NLR (continuous) | 1.00 (0.98–1.03) | 0.799 | 1.01 (0.98–1.03) | 0.628 |
| Albumin (continuous) | 0.44 (0.26–0.74) | 0.002 | 0.33 (0.20–0.55) | <0.001 |
| SMI (continuous) | 0.98 (0.95–1.00) | 0.061 | 0.97 (0.94–1.00) | 0.049 |
| BMI (continuous) | 0.99 (0.97–1.01) | 0.344 | 0.97 (0.92–1.02) | 0.232 |
| MMR (continuous) | 10.03(0.35–288.91) | 0.179 | 48.30 (2.02–1157.87) | 0.017 |
| Composite score (continuous) | 1.51 (1.17–1.93) | 0.001 | 1.93 (1.46–2.54) | <0.001 |

HR indicates hazard ratio and CI indicates confidence interval.
The observed association between MMR and overall survival should be interpreted with caution due to the extremely wide confidence intervals, suggesting limited precision and potential influence of outliers.

**Supplementary Table 5.** Model comparison analyses for progression-free and overall survival.

| Outcome | Model | C-index | AIC |
| --- | --- | --- | --- |
| PFS | Clinical model only | 0.615 | 726.6 |
| PFS | Clinical model + high NLR | 0.638 | 723.0 |
| PFS | Clinical model + albumin <3.5 g/dL | 0.658 | 712.3 |
| PFS | Clinical model + low SMI | 0.634 | 728.2 |
| PFS | Clinical model + albumin–SMI score | 0.650 | 721.4 |
| PFS | Clinical model + composite score, 0–3 | **0.659** | 716.2 |
| PFS | Clinical model + composite risk group, 2–3 vs 0–1 | 0.651 | 719.0 |
| OS | Clinical model only | 0.607 | 606.7 |
| OS | Clinical model + high NLR | 0.694 | 594.9 |
| OS | Clinical model + albumin <3.5 g/dL | 0.671 | 594.4 |
| OS | Clinical model + low SMI | 0.658 | 606.0 |
| OS | Clinical model + albumin–SMI score | 0.678 | 596.4 |
| OS | Clinical model + composite score, 0–3 | **0.717** | **585.2** |
| OS | Clinical model + composite risk group, high vs low | 0.689 | 595.9 |

The clinical model included age ≥65 years, ECOG performance status ≥2, and liver metastasis. The albumin–SMI score included hypoalbuminemia and low SMI as two binary components. The composite score was evaluated as an ordinal variable ranging from 0 to 3. Higher C-index and lower AIC values indicate better prognostic discrimination and model fit, respectively.

**Supplementary Table 6.** Sensitivity analysis restricted to patients with available PD-L1 expression data.

| Outcome | Model | HR (95% CI) | p value |
| --- | --- | --- | --- |
| PFS | Univariable composite risk group, high vs low | 2.22 (1.23–4.01) | 0.008 |
| PFS | Adjusted composite risk group, high vs low | 2.71 (1.46–5.04) | 0.002 |
| OS | Univariable composite risk group, high vs low | 2.16 (1.11–4.20) | 0.024 |
| OS | Adjusted composite risk group, high vs low | 2.78 (1.37–5.64) | 0.005 |

The analysis included patients with available PD-L1 expression data (n = 60). Adjusted models included age ≥65 years, ECOG performance status ≥2, liver metastasis, and composite risk group.

**Supplementary Table 7.** Sensitivity analyses evaluating individual host-related parameters, the NLR-free score, and BMI-integrated SMI classification.

| Analysis | PFS adjusted  HR (95% CI) | p value | OS adjusted  HR (95% CI) | p value |
| --- | --- | --- | --- | --- |
| High NLR | 1.66 (1.09–2.52) | 0.018 | 2.39 (1.50–3.80) | <0.001 |
| Albumin <3.5 g/dL | 3.52 (2.03–6.10) | <0.001 | 3.42 (1.93–6.03) | <0.001 |
| Low SMI | 1.15 (0.76–1.74) | 0.523 | 1.46 (0.92–2.31) | 0.105 |
| Composite risk group, high vs low | 2.05 (1.32–3.19) | 0.001 | 2.48 (1.54–4.01) | <0.001 |
| Composite score, per 1-point increase | 1.62 (1.24–2.11) | <0.001 | 2.02 (1.52–2.68) | <0.001 |
| NLR-free albumin–SMI score, per 1-point increase | 1.64 (1.14–2.35) | 0.007 | 2.03 (1.37–3.01) | <0.001 |
| BMI-integrated composite score, per 1-point increase | 1.72 (1.29–2.29) | <0.001 | 2.04 (1.51–2.75) | <0.001 |

Adjusted models included age ≥65 years, ECOG performance status ≥2, liver metastasis, and the evaluated host-related variable. Individual host-related parameters were entered into separate adjusted models. The NLR-free albumin–SMI score included hypoalbuminemia and low SMI as two binary components. The BMI-integrated composite score used BMI-adjusted SMI classification instead of the primary sex-specific SMI definition.

**Supplementary Table 8.** Interobserver reproducibility analysis for skeletal muscle index measurement.

| Parameter | Result |
| --- | --- |
| Number of patients assessed | 30 |
| Reader 1 SMI, mean ± SD, cm²/m² | 47.95 ± 8.22 |
| Reader 2 SMI, mean ± SD, cm²/m² | 47.03 ± 8.29 |
| Mean difference, cm²/m² | −0.91 |
| Pearson correlation coefficient | 0.978 |
| Intraclass correlation coefficient | 0.973 |

SMI, skeletal muscle index; SD, standard deviation. Interobserver reproducibility was assessed in a randomly selected subset of 30 patients using the intraclass correlation coefficient based on a two-way random-effects model for absolute agreement.

**Supplementary Table 9.** Distribution of nivolumab-related adverse events

| **Adverse event** | **Any grade  n (%)** | **Grade ≥3  n (%)** |
| --- | --- | --- |
| Pneumonitis | 5 (4.5) | 2 (1.8) |
| Hypothyroidism | 2 (1.8) | 0 |
| Colitis | 2 (1.8) | 1 (0.9) |
| Skin toxicity* | 3 (2.7) | 1 (0.9) |
| Pruritus | 1 (0.9) | 0 |
| Hepatitis | 1 (0.9) | 0 |
| Immune neuritis | 1 (0.9) | 1 (0.9) |
| Demyelinating polyneuropathy | 1 (0.9) | 1 (0.9) |

*****Skin toxicity includes rash and lichenoid dermatitis.

**Supplementary Figure 1.** Kaplan–Meier curves for progression-free survival according to skeletal muscle index status.

Low SMI was defined as <52 cm²/m² for men and <38 cm²/m² for women.

**Supplementary Figure 2.** Kaplan–Meier curves for overall survival according to neutrophil-to-lymphocyte ratio (NLR).

Patients were stratified according to the cohort median NLR value of 3.76.

**Supplementary Figure 3.** Kaplan–Meier curves for overall survival according to serum albumin level.

Patients were stratified according to albumin level (<3.5 g/dL vs ≥3.5 g/dL).

**Supplementary Figure 4.** Kaplan–Meier curves for overall survival according to metabolic muscle ratio (MMR).

Patients were stratified according to the median metabolic muscle ratio of the study cohort.

**Supplementary Figure 5.** Kaplan–Meier curves for overall survival according to the composite inflammatory–nutritional– skeletal muscle score.

The composite score ranged from 0 to 3, with higher scores indicating greater inflammatory, nutritional, and skeletal muscle burden. Patients were categorized into lower-risk (score 0–1) and higher-risk (score 2–3) groups for survival analyses.

**Supplementary Methods: PET/CT Acquisition and Quantitative Analysis**

PET/CT imaging was performed according to standardized institutional protocols. Patients fasted for at least 6 hours prior to imaging, and blood glucose levels were confirmed to be <200 mg/dL before tracer administration. A weight-adjusted dose of ^18F-FDG (3.5–5.0 MBq/kg) was administered intravenously, followed by an uptake period of approximately 50–60 minutes. Imaging was acquired from the skull vertex to the mid-thigh using dedicated PET/CT systems (Discovery 710, GE Healthcare, Milwaukee, WI, USA).

Image reconstruction was performed using an ordered subset expectation maximization (OSEM) algorithm (3 iterations, 24 subsets) with a 256 × 256 matrix and a 5-mm Gaussian filter.

For metabolic muscle assessment, axial fused PET/CT images at the level of the third lumbar vertebra (L3) were used. Three-dimensional spherical volumes of interest (VOIs; 1 cm³) were manually placed within both right and left psoas muscles. For each muscle, three VOIs were positioned within the muscle parenchyma while avoiding adjacent structures such as bowel loops, ureters, and adipose tissue.

SUVmean values were recorded from each VOI, and the average of all VOIs from both psoas muscles was used to represent psoas muscle metabolic activity. For liver reference, a VOI was placed in the right hepatic lobe, and liver SUVmean was recorded. The metabolic muscle ratio (MMR) was defined as the ratio of psoas muscle SUVmean to liver SUVmean.

All imaging analyses were performed by investigators experienced in oncologic PET/CT interpretation.

**List of Abbreviations**

BMI — Body mass index

CI — Confidence interval

ECOG — Eastern Cooperative Oncology Group

HR — Hazard ratio

IQR — Interquartile range

MMR — Metabolic muscle ratio

NLR — Neutrophil-to-lymphocyte ratio

OS — Overall survival

PFS — Progression-free survival

SMI — Skeletal muscle index
